# Advancements and progress in juvenile idiopathic arthritis: A Review of pathophysiology and treatment

**DOI:** 10.1097/MD.0000000000037567

**Published:** 2024-03-29

**Authors:** Helen Ye Rim Huang, Andrew Awuah Wireko, Goshen David Miteu, Adan Khan, Sakshi Roy, Tomas Ferreira, Tulika Garg, Narjiss Aji, Faaraea Haroon, Farida Zakariya, Yasir Alshareefy, Anushka Gurunath Pujari, Djabir Madani, Marios Papadakis

**Affiliations:** a Faculty of Medicine and Health Science, Royal College of Surgeons in Ireland, Dublin, Ireland; b Sumy State University, Sumy, Ukraine; c School of Biosciences, Biotechnology, University of Nottingham, Nottingham, UK; d Department of Biochemistry, Caleb University Lagos, Lagos, Nigeria; e Kent and Medway Medical School, Canterbury, Kent, UK; f School of Medicine, Queen’s University Belfast, Belfast, Northern Ireland, UK; g School of Clinical Medicine, University of Cambridge, Cambridge, UK; h Government Medical College and Hospital Chandigarh, Chandigarh, India; i Faculty of Medicine and Pharmacy of Rabat, Rabat, Morocco; j Faculty of Public Health, Health Services Academy, Islamabad, Pakistan; k Faculty of Pharmaceutical Sciences, Ahmadu Bello University Zaria, Zaria, Nigeria; l School of Medicine, Trinity College Dublin, The University of Dublin, Dublin, Ireland; m Department of Kinesiology, Faculty of Science, McMaster University, Hamilton, Ontario, Canada; n UCD Lochlann Quinn School of Business and Sutherland School of Law, University College Dublin, Dublin, Ireland; o Department of Surgery II, University Hospital Witten-Herdecke, University of Witten-Herdecke, Wuppertal, Germany.

**Keywords:** genetics, immunology, juvenile idiopathic arthritis, rheumatology, risk stratification, treatment

## Abstract

Juvenile idiopathic arthritis (JIA) is a chronic clinical condition characterized by arthritic features in children under the age of 16, with at least 6 weeks of active symptoms. The etiology of JIA remains unknown, and it is associated with prolonged synovial inflammation and structural joint damage influenced by environmental and genetic factors. This review aims to enhance the understanding of JIA by comprehensively analyzing relevant literature. The focus lies on current diagnostic and therapeutic approaches and investigations into the pathoaetiologies using diverse research modalities, including in vivo animal models and large-scale genome-wide studies. We aim to elucidate the multifactorial nature of JIA with a strong focus towards genetic predilection, while proposing potential strategies to improve therapeutic outcomes and enhance diagnostic risk stratification in light of recent advancements. This review underscores the need for further research due to the idiopathic nature of JIA, its heterogeneous phenotype, and the challenges associated with biomarkers and diagnostic criteria. Ultimately, this contribution seeks to advance the knowledge and promote effective management strategies in JIA.

## 1. Introduction

Juvenile idiopathic arthritis (JIA) is a chronic rheumatic condition characterized by prolonged synovial inflammation, which can lead to structural joint damage and extra-articular organ involvement.^[1]^ The chronic nature of JIA and its potential for irreversible complications significantly impact the quality of life for affected individuals and their families. Despite being one of the most common chronic rheumatic conditions in pediatrics, the phenotypic variability of JIA often results in misdiagnosis or underdiagnosis, highlighting the need for a better understanding of its etiology.

JIA is a global health concern, impacting an estimated 3 million children and young adults worldwide.^[2]^ Extensive research conducted in high-income countries, as reported by Ravelli et al in 2007, has revealed prevalence rates of JIA ranging from 16 to 150 cases per 100,000 people.^[3]^ However, a recent study focusing on Africa and the Middle East identified lower prevalence rates in these regions, ranging from <3.43 to <22 cases per 100,000 population.^[2]^ Notably, gender disparities persist, with girls consistently facing a higher risk of JIA compared to boys and the oligoarticular subtype being predominant.^[2]^ Interestingly, the subtype distribution varies across different regions, with Western European countries commonly experiencing oligoarthritis prevalence, while countries like Costa Rica, India, New Zealand, and South Africa exhibit a predominance of the polyarthritis subtype.^[3]^

The pathophysiology of JIA remains idiopathic, impeding the development of a definitive diagnostic algorithm and the identification of molecular biomarkers that could aid in early diagnosis.^[1,4]^ To address this challenge, it is crucial to apply evidence-based decision-making and leverage the potential of translational medicine to enhance therapeutic pathways and improve diagnostics.

An accurate diagnosis of JIA relies on understanding its distinct subtypes. Oligoarticular JIA, involving a limited number of joints, presents with redness, swelling, and a limited range of motion, while uveitis can also occur.^[5]^ Polyarticular JIA affects more than 4 joints within the first 6 months, with variations based on rheumatoid factor presence.^[6]^ Differential diagnoses must consider similar clinical features seen in infections or malignancies.

This review critically evaluates the existing literature on JIA, identifies research gaps, and proposes recommendations for future investigations. By synthesizing available evidence, we shed light on JIA’s multifactorial nature, enhancing therapeutic outcomes and diagnostic risk stratification through innovative advancements. We explore various studies, including in vivo animal models and large-scale genome-wide analyses, to uncover key mechanisms in JIA pathogenesis. This knowledge facilitates targeted therapies and personalized treatment approaches. By delving into JIA diagnostics and therapeutics, this review consolidates existing knowledge, offering insights for further research.

## 2. Methodology

A comprehensive literature review was conducted to examine the pathophysiology and treatment of JIA. The search encompassed multiple electronic databases, including PubMed, MEDLINE, Embase, and Cochrane Library, using the following keywords: “juvenile idiopathic arthritis,” “JIA,” “pathophysiology,” “treatment,” “translational medicine.” The search was limited to articles published between [1996] and [2023].

Inclusion criteria encompassed studies focusing on the pathophysiology or treatment of JIA, involving human subjects or relevant animal models, and providing insights into translational research. Studies reporting original research findings, systematic reviews, meta-analyses, or clinical trials were included. Furthermore, articles had to be published in English. Exclusion criteria were applied to studies unrelated to JIA, those lacking primary research findings or relevant clinical information, and articles published in languages other than English. To ensure the accuracy and consistency of the selection process, 2 independent reviewers (H.H) and (G.M) assessed the relevance of titles and abstracts. Full-text articles were retrieved for further evaluation. In cases where consensus was not reached, a third author (W.A.A) was consulted to make a final decision.

The findings from the selected studies were synthesized in a narrative format, focusing on key themes related to JIA pathophysiology, treatment approaches, and advancements. The results were organized to address the specific objectives of the review. Additionally, key findings, knowledge gaps, and future research directions were discussed, providing a comprehensive overview of the current state of knowledge in the field of JIA. A summary of the methodology employed is presented in Table 1.

**Table 1:** Summary of methodology for this review.

Methodology step	Description
Literature search database	PubMed, SCOPUS, EMBASE, Cochrane Library
Inclusion criteria	Full-text articles published between 1996 and 2023 such as original clinical and laboratory research studies systematic reviews, meta-analyses, or clinical trials, with specific restrictions to the English Language. A diverse range of studies were included such as case-control studies, cross-sectional studies, retrospective or prospective cohort studies, randomized controlled trials, systematic reviews, meta-analyses, and laboratory research studies including in vivo and in vitro models. Studies discussing JIA in pediatric populations were screened for relevant information and summarized in a narrative fashion.
Exclusion criteria	Stand-alone abstracts, those lacking relevant clinical information, and studies that are not published in English.
Search terms	Precise terms such as “juvenile idiopathic arthritis,” “JIA,” “pathophysiology,” “treatment,” “translational medicine” was conducted between [1996] and [2023].
Sample size	No strict sample size required.

## 3. Results and discussion

### 3.1. Clinical phenotypes, differentials, and classification systems

#### 3.1.1. Introduction to clinical phenotypes.

**Figure 1.**
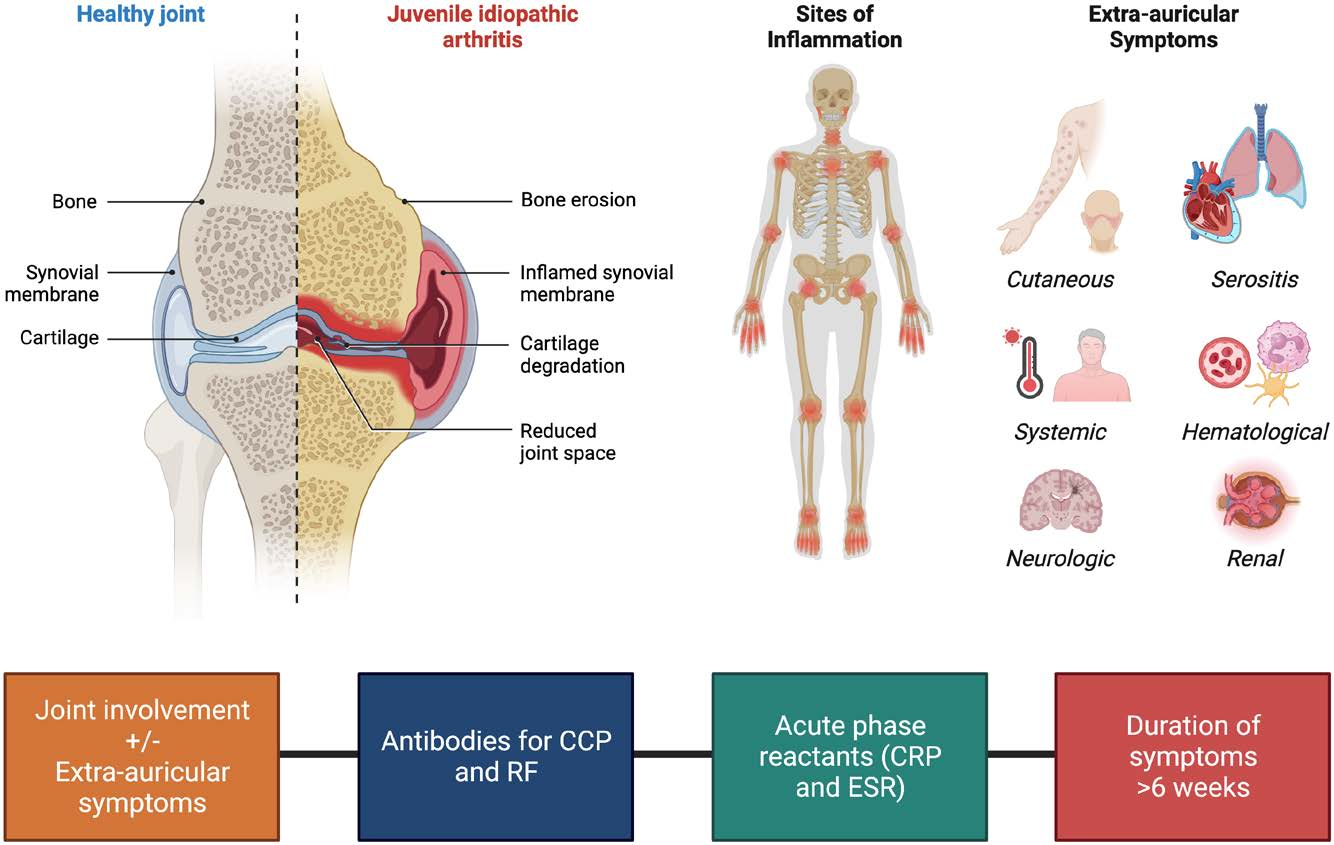
Clinical features of juvenile idiopathic arthritis. By BioRender.com (2022). Anti-CCP = anti-cyclic citrullinated protein; CRP = c-reactive protein; ESR = erythrocyte sedimentation rate; RF = rheumatoid factor.

JIA is a heterogeneous condition characterized by inflammation in at least one joint in individuals younger than 16 years old, with symptoms persisting for a minimum of 6 weeks. Key features of JIA include stiffness in the affected joints during the early hours of the day or following prolonged inactivity, limitations in the use of affected joints during daily activities, and the presence or absence of pain (Fig. 1).^[1,7]^ While these general characteristics form the basis for JIA diagnosis, it is important to recognize that the condition encompasses diverse clinical presentations or phenotypes. These distinct phenotypes capture the variability in symptoms, disease progression, and joint involvement among individuals with JIA.

#### 3.1.2. Classification systems.

##### 3.1.2.1. International league of associations for rheumatology (ILAR) classification.

The ILAR classification system is widely used to categorize JIA into several subtypes based on specific clinical features and disease duration. The ILAR criteria help in determining the appropriate subtype for a given JIA case. The subtypes recognized within the ILAR classification include systemic arthritis, oligoarthritis, polyarthritis, enthesitis-related arthritis, psoriatic arthritis, and undifferentiated arthritis.^[1]^

The ILAR classification system employs various criteria to differentiate between the subtypes, including the number of affected joints, the presence of systemic symptoms such as fever and rash, and laboratory findings such as elevated inflammatory markers.^[1]^ Each subtype within the ILAR classification exhibits distinct clinical characteristics.

##### 3.1.2.2. Other classification systems.

In addition to the ILAR classification, other classification systems have been used in the evaluation and categorization of JIA. Examples include the Wallace criteria and the American College of Rheumatology provisional criteria.^[8]^ These alternative classification systems may differ in their approach and criteria for subtype determination.

A comparison between these alternative classification systems and the ILAR criteria reveals both strengths and limitations. While the ILAR classification is widely accepted and valuable for research purposes, it has faced criticism for not adequately distinguishing between forms of chronic arthritis observed in both children and adults.^[6]^ Consequently, the diagnosis of JIA still relies on excluding other potential causes, and definitive diagnostic tests for JIA remain unavailable.^[9]^

#### 3.1.3. Clinical features and phenotypes.

##### 3.1.3.1. Oligoarticular JIA.

Oligoarticular JIA is characterized by the involvement of a limited number of joints, typically 4 or fewer, particularly those in the extremities. In this subtype, joint inflammation often follows an asymmetric pattern, with affected joints presenting with features such as redness, swelling, and a restricted range of motion. Notably, uveitis, inflammation of the eye, can also occur in individuals with oligoarticular JIA.^[10]^ Prognostically, oligoarticular JIA has variable outcomes, with some cases following a self-limiting course while others may progress to a chronic form. It is important to consider differential diagnoses such as infections or malignancies that may present with similar clinical features when evaluating a patient with oligoarticular JIA.

##### 3.1.3.2. Polyarticular JIA.

Polyarticular JIA is characterized by inflammation involving more than 4 joints within the first 6 months following diagnosis.^[6]^ This subtype can be further divided into rheumatoid factor-positive or negative polyarthritis. In rheumatoid factor-negative polyarticular JIA, joint inflammation follows an asymmetric pattern, while in rheumatoid factor-positive polyarticular JIA, joint involvement occurs symmetrically. Polyarticular JIA shares clinical characteristics with adult rheumatoid arthritis, such as joint swelling, morning stiffness, and systemic symptoms.^[6]^

To differentiate between JIA and conditions with similar phenotypes, it is crucial to consider differential diagnoses, including malignancies and infection-related arthritis. Malignancies can present with clinical features similar to JIA, but accompanying hematologic changes specific to malignancies help differentiate them. Infection-related arthritis, such as pyogenic arthritis, reactive arthritis, septic arthritis, and postinfection arthritis, can also manifest with symptoms resembling JIA, particularly in individuals under 16 years old.^[9]^ Distinguishing criteria between infection-related arthritis and JIA include isolating and culturing the implicated microorganism from the joint and assessing for progressive fever and malaise, which are indicative of an infection.^[2]^

To establish a definitive diagnosis, physical examination, medical history assessment, and laboratory tests are employed. While physical examination and medical history help assess the family history, number of affected joints, severity of the condition, and systemic involvement, they are not definitive on their own. Laboratory tests for JIA include antinuclear antibody tests, autoantibody tests, assessment of inflammatory markers such as erythrocyte sedimentation rate and C-reactive protein, rheumatoid factor and anti-cyclic citrullinated peptide antibody testing, complete blood count, and human leukocyte antigen-B27 (HLA-B27) gene panel.^[11]^ However, these tests lack specificity for JIA, and their presence, absence, or abnormalities do not definitively indicate the presence of JIA. Imaging techniques can also be used to examine bone structures, joint damage, fluid accumulation, and inflammation in specific bones, cartilage, and joints. However, it is important to note that imaging findings may often be incidental and nonspecific. Other forms of imaging are not currently considered in the diagnostic algorithm for JIA, highlighting the need for further research to address this gap in clinical practice.^[11]^

### 3.2. Pathophysiology of JIA

**Figure 2.**
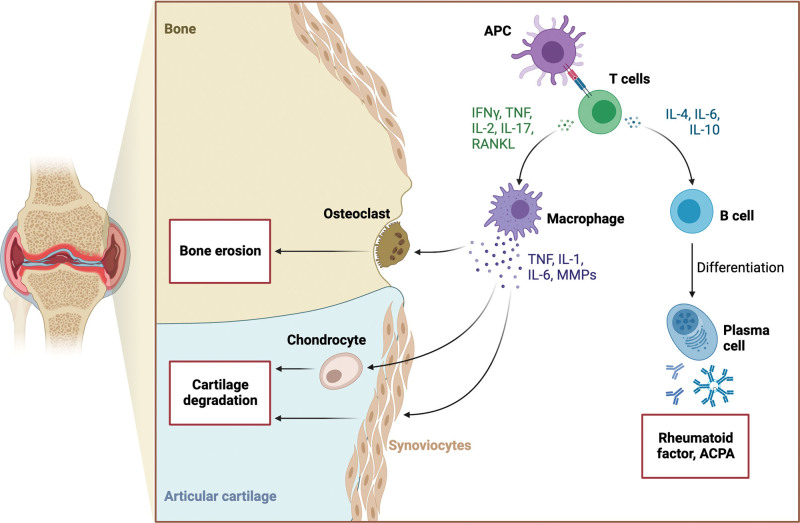
Pathophysiology and immune processes for juvenile idiopathic arthritis. Adapted from “Pathogenesis of Rheumatoid Arthritis II’’ by BioRender.com (2022). Retrieved from https://app.biorender.com/biorender-templates. ACPA = anti-citrullinated protein antigen; APC = antigen-presenting cells; IFNγ = interferon gamma; IL = interleukin; RANKL = receptor activator of nuclear factor kappa-β ligand; TNF = tumor necrosis factor.

The pathophysiology of JIA involves an autoimmune response where the body’s immune system attacks its own cells. This process is driven by interactions among immune cells, such as lymphocytes, monocytes, macrophages, and neutrophils, along with pro-inflammatory cytokines like interleukins (IL) as shown in Figure 2. These interactions lead to an inflammatory response characteristic of JIA.^[1]^

The exact triggers and predispositions for this inflammatory response, influenced by environmental and genetic factors, are not fully understood.^[4]^ Different immunological changes occur in various JIA subtypes, including abnormal immune cell activation, T cell differentiation producing pro-inflammatory cytokines, involvement of the innate immune system, enthesitis, adaptive immune mechanisms, and autoinflammatory pathology. Systemic JIA, often associated with macrophage activation syndrome (MAS), is particularly influenced by these processes.^[4]^ Serological biomarkers, mainly inflammatory cytokines, have shown promise in detecting and diagnosing JIA, providing insights into its chronic progression.^[12]^ Notably, patients positive for both anti-cyclic citrullinated protein and rheumatoid factor exhibit higher levels of tumor necrosis factor alpha (TNFɑ), IL1β, IL-6, and IL-17.^[11,13]^

Animal models are employed to gain further understanding of the idiopathic nature of JIA. Studies on interferon-gamma (INF*γ*), IL-6, IL-10, and IL-17 have revealed insights. Injection of complete Freund adjuvant in wild-type (WT) and INF*γ*-knockout (KO) mice led to increased cytokine production, particularly IL-6 and IL-17 in INF*γ*-KO mice, produced by CD-4 + and innate T cells.^[14]^ In INF-*γ* KO mice, defective IL-10 production by CD4 + regulatory T cells, CD19 + B cells, and natural killer (NK) cells was observed. B cells were identified as the major source of IL-10. Similar findings were observed in systemic juvenile idiopathic arthritis (sJIA) patients, implicating IL-10 in JIA pathogenesis. Neutralization of IL-10 in colonization factor antigen-challenged WT mice resulted in the transition from a transient inflammatory process to chronic disease.^[15,16]^ In another sJIA mouse model, the regulatory role of NK cells was evident. Depletion of NK cells or blockade of natural killer group 2, member D (NKG2D), an NK cell activating receptor, increased systemic inflammation and sJIA symptoms in WT mice. Conversely, INF-*γ* KO mice exhibited defective degranulation capacities of NK cells against activated immune cells, contributing to sJIA development.^[17]^

JIA subtypes and evolving terminology contribute to the disease’s heterogeneity, making it difficult to determine the precise role of environmental factors. Infections, genetic susceptibility, stress, and maternal smoking are commonly associated with JIA. Cohort studies have replicated associations between JIA and specific histocompatibility genetic variants, although the strength of the association varies by subtype. For instance, rheumatoid factor + JIA is linked to HLA DR4, oligoarthritis to HLA DR5, HLA DR8, and HLA DR11, enthesitis-related arthritis to HLA B27, and psoriatic arthritis to HLA DR1 and HLA DR6. Moreover, a multi-ethnic study revealed moderately increased risks for JIA patients of European descent compared to those of African, Asian, or Indian descent.^[18]^

### 3.3. Genetic contributions to disease progression and pathology

#### 3.3.1. Monogenic variants identification with multi-omics.

Multi-omics investigations have significantly contributed to the study of genetic and molecular mechanisms involved in the pathogenesis of JIA. Although only a small number of familial JIA cases can be attributed to single-gene mutations, the majority of sporadic cases are influenced by genetic, epigenetic, and environmental factors. Despite the classification of JIA into 7 categories, there is phenotypic overlap among subtypes, suggesting a common genetic or epigenetic basis. The identification of monogenic variants of JIA has significantly improved our understanding of key molecular pathways, which is particularly important given the poorly understood causes of JIA and the ambiguities in its classification (see Table 2 for more information).

**Table 2:** Genes associated with juvenile idiopathic arthritis’s monogenic forms.

Genes	Causal mutations	Related subtype of JIA	Functional evidence	Mechanism	Citations
LACC1	p.Cys284Arg, p.ILe254Val, rs3816311, p.Arg414Ter, p.Ile330del, p.CYs43Tyrfs*6	Systemic JIA	The TNF levels in LACC1/mice were elevated. In macrophages and dendritic cells, LPS and other TLR ligands increased LACC1 transcripts and protein levels	Regulation of inflammation	^[19–22]^
LRBRA	–	Oligoarthritis	High quantities of serum and secretory IgA are produced by LRBA/mice	Peripheral tolerance issues	^[23]^
UNCD13	c.117 + 143A>G 753 + 3 [G>A], 1579 [C>T] R527W	Systemic JIA	Human effector CD8 + T lymphocytes and developed NK cells both showed high levels of Munc13-4 expression. Upon cytotoxic lymphocyte differentiation, the expression of Munc13-4 was specifically increased	Disrupting the binding of transcription factors	^[24–26]^
NFIL3	p.M170I	Systemic JIA	Mutations in NFIL3 cause increased IL-1	Irritation for arthritis. IL-1 overproduction during innate immune system development	^[27]^

IgA = immunoglobulin A, IL-1 = interleukin 1, JIA = juvenile idiopathic arthritis, LACC1 = laccase domain containing 1, LPS = lipopolysaccharide, LRBA = LPS-responsive beige-like anchor protein, MUNC13-4 = mammalian Unc-13 homolog D, NFIL3 = nuclear factor, interleukin 3 regulated, NK = natural killer cells, TLR = Toll-like receptor, TNF = tumour necrosis factor, UNC13 = unc-13 homolog.

Skon-Hegg et al (2019) recently demonstrated the crucial role of laccase domain containing 1 (LACC1) in inflammatory responses through in vivo functional research. By comparing LACC1 knockout mice to WT mice they observed worsened disease in models of collagen-induced arthritis and Citrobacter rodentium-induced colitis.^[19]^ Additionally, the harmful effects of the LACC1 mutation I254V have been associated with other conditions beyond JIA, such as inflammatory bowel disease, leprosy, and Behçet disease.^[20]^ Furthermore, a Saudi family exhibited an association between the C284R mutation and severe pediatric Crohn disease.^[21]^

The WDL-BEACH-WD gene family includes the lipopolysaccharide (LPS) responsive beige-like anchor protein gene, which is upregulated in macrophages and B-cells in response to bacterial LPS.^[22]^ LPS-Responsive Beige-like Anchor protein prevents the degradation of cytotoxic T-lymphocyte-associated antigen 4 (CTLA-4) and maintains its intracellular storage.^[23]^ Upon antigen recognition, the T cell receptor (TCR) complex requires CD28 co-stimulation for T-lymphocyte activation.^[24]^ CTLA-4, by competing with CD28 for the B7 ligands, transmits inhibitory signals to reduce T cell activation.^[24]^ Reduced CTLA-4 activity has been implicated in JIA.^[25]^ Interestingly, JIA patients exhibit higher levels of CTLA-4 expression on their CD4+ CD28− T cells, and CD28-T cells demonstrate resistance to CTLA-4 inhibition.^[26]^

Intronic mutation c.117 + 143A>G in unc-13 homolog D (UNC13D) has been linked to systemic JIA and recurrent macrophage activation syndrome (MAS) in a patient, as discovered by Schulert et al (2018).^[27]^ This mutation disrupts the interaction of NF-interaction B with a transcriptional enhancer, leading to downregulated UNC13D transcription in peripheral blood mononuclear cells. Previous investigations have also revealed a genetic association between sJIA/MAS and sequence polymorphisms in Munc13-4 (UNC13D).^[28]^ Hazen et al (2008) identified compound heterozygous mutations of UNC13D in an 8-year-old girl with systemic JIA but without MAS, which resulted in reduced NK cell cytotoxicity.^[29]^

Nuclear factor interleukin 3 regulated gene (NFIL3) is an essential transcription factor in the immune system that regulates the cytokine production of type 2 T helper (T(H)2) cells.^[30]^ Lower levels of NFIL3 expression have been observed in patients with Crohn disease and ulcerative colitis, potentially leading to proinflammatory roles of macrophages instead of tolerogenic roles.^[31]^ Schlenner et al conducted flow cytometry, single-cell sequencing, and whole-exome sequencing analyses on monozygotic twin girls with JIA.^[32]^ These patients harbored a novel homozygous mutation (M170I) in NFIL3, which destabilized the NFIL3 protein. Nfil3-knockout mice showed increased IL-1 production and were more susceptible to developing arthritis.^[32]^

#### 3.3.2. Large-scale genotype–phenotype association studies.

Large-scale genotype–phenotype association studies have played a vital role in identifying genetic variants associated with JIA, enabling a deeper understanding of its etiology and pathogenesis. Various genetic study approaches, including genome-wide association studies (GWAS), next-generation sequencing, electronic health record (EHR) studies, phenome-wide association studies (PheWAS), mendelian randomization (MR) associations, and linkage studies, have significantly contributed to our knowledge of JIA.

GWAS studies have identified multiple genetic loci that have been implicated in the pathogenesis of JIA. Genes such as protein tyrosine phosphatase non-receptor type 2 (PTPN2), protein tyrosine phosphatase non-receptor type 22 (PTPN22), TNF alpha induced protein 3 (TNFAIP3), and interleukin 2 receptor subunit alpha (IL2RA), involved in immune responses, have been reported to cause JIA.^[33–36]^ Furthermore, GWAS studies have identified genes regulating cartilage and bone growth, including interleukin 6 receptor (IL6R), SH2B adaptor protein 3 (SH2B3), and juxtaposed with another zinc finger 1 (JAZF1).^[37–39]^ Complementing these findings, EHR studies have leveraged large-scale patient data, including genetic information, to identify genetic risk loci for JIA. These studies have confirmed previously identified associations and discovered new genetic associations.^[40–42]^ Next-generation sequencing studies have further expanded our understanding by providing insights into the genetic architecture of JIA through genomic profiling and the identification of additional genetic risk loci.^[43]^ By analyzing protein-coding regions of the genome in JIA patients compared to controls, rare genetic variants affecting immune cell function and inflammatory molecule production have been identified.^[44,45]^ Additionally, copy number variations and structural variations have been detected in JIA patients, which are not captured by GWAS and EHR studies. PheWAS studies have investigated genetic associations with specific JIA subtypes and phenotypic characteristics such as joint involvement and the presence of specific autoantibodies. These studies have revealed common genetic risk loci for oligoarticular JIA, polyarticular JIA, and psoriatic JIA.^[46,47]^ Mendelian randomization studies have provided insights into identifying genetically causative variants for JIA, further unraveling the mechanisms underlying JIA susceptibility and associated outcomes. Moreover, MR studies have helped in understanding racial, ethnic, and geographical variations in JIA.^[21,48–50]^ Linkage studies have focused on families with multiple affected members to identify genetic risk loci or regions for JIA, contributing to the identification of genetic regions of interest.^[51–53]^

The interdisciplinary use of genetic association studies has provided a comprehensive overview of the pathogenesis and potential treatments for different JIA subtypes by comparing the genotypes of affected patients to healthy groups. PheWAS has identified multiple diseases and traits that might have been missed due to small sample sizes, shedding light on the underlying pathways involved. Linkage studies have demystified regions in the genome responsible for JIA within families with multiple affected individuals. Mendelian randomization studies have effectively used known genetic variants as instrumental variables to infer causative relationships and outcomes in JIA patients.

### 3.4. Treatment guidelines: indications and draw-backs

#### 3.4.1. Overview.

Treatment guidelines play a critical role in managing juvenile idiopathic arthritis (JIA) by offering evidence-based recommendations for healthcare professionals. These guidelines ensure standardized and optimal care, leading to improved patient outcomes and enhanced quality of life. Notably, advancements in treatment options have significantly enhanced the prognosis of JIA, resulting in reduced pain and disability.^[5]^ The primary objectives of JIA treatment encompass alleviating symptoms, preventing joint damage, and enhancing immune function and overall well-being.^[5]^ Individualized management of JIA is based on specific symptoms, utilizing diverse treatment modalities such as medications, physical therapy, and occupational therapy. Physical therapy aims to enhance joint mobility and muscle strength, while occupational therapy educates patients on minimizing joint stress during daily activities.^[54]^

In both the United States and Europe, a stepwise approach is commonly employed for JIA management. It commences with nonsteroidal anti-inflammatory drugs (NSAIDs) and escalates to more aggressive therapies when necessary.^[55]^ The European League Against Rhematism recommends Janus kinase (JAK) inhibitors, a novel class of targeted therapies, as effective treatments for JIA. However, caution must be exercised as the use of JAK inhibitors is associated with an increased risk of serious infections, malignancies, and blood clots.^[55,56]^ These guidelines undergo regular updates to integrate new evidence and advancements in the field.

#### 3.4.2. Pharmacological Interventions.

**Figure 3.**
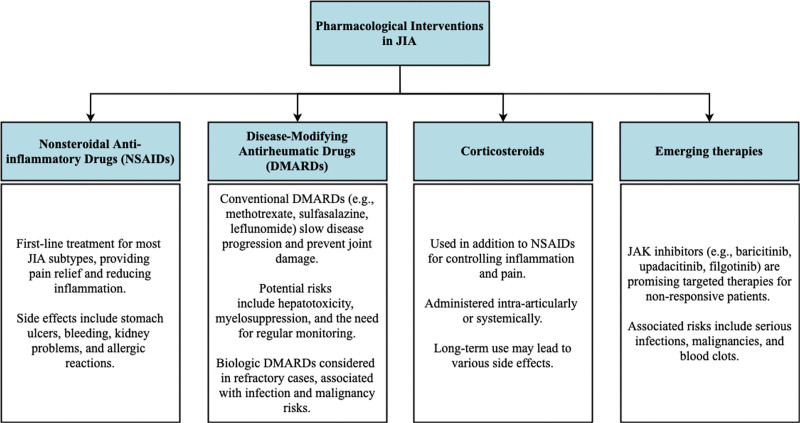
Pharmacological interventions in JIA. JAK = janus kinase; JIA = juvenile idiopathic arthritis.

Treatment guidelines encompass a range of pharmacological interventions that have demonstrated efficacy in managing JIA. These interventions are categorized into different classes, each with its own indications, mechanisms of action, and potential drawbacks. It is important for healthcare professionals to be aware of these interventions to make informed decisions regarding their prescription. Figure 3 summarizes the pharmacological interventions widely used in JIA.

##### 3.4.2.1. Nonsteroidal anti-inflammatory drugs.

For most JIA subtypes, first-line treatment involves the use of NSAIDs, such as Ibuprofen and naproxen, due to their ability to alleviate pain and reduce inflammation. They are effective in managing mild to moderate disease activity, but their use is limited to symptom relief and does not address the underlying disease process. However, these drugs carry the risk of side effects, including stomach ulcers, bleeding, and kidney problems.^[56]^ Allergic reactions have also been reported in some patients. Therefore, close monitoring of patients using NSAIDs is essential to manage and minimize these risks.

##### 3.4.2.2. Disease-modifying antirheumatic drugs (DMARDs).

DMARDs are another class of medications employed in the treatment of JIA. Conventional DMARDs, such as methotrexate, sulfasalazine, or leflunomide, are used to slow the progression of the disease and prevent joint damage.^[57]^ DMARDs are a key component of JIA treatment, particularly in cases where NSAIDs alone are insufficient. DMARDs aim to suppress the immune system and modify the disease course, thereby reducing joint damage and improving long-term outcomes. Methotrexate is the most commonly prescribed DMARD for JIA, but other options such as sulfasalazine and leflunomide may also be considered. However, DMARDs carry potential risks, including hepatotoxicity, myelosuppression, and the need for regular monitoring. Biologic DMARDs, including TNF inhibitors (e.g., etanercept, adalimumab, tocilizumab, and infliximab) and JAK inhibitors (e.g., baricitinib, upadacitinib, and filgotinib), may also be considered in cases where the disease is refractory to treatment with other medications.^[58]^ Biologic agents target specific components of the immune system involved in the inflammatory response of JIA, TNF inhibitors have shown efficacy in ameliorating JIA conditions. It is important to note that the use of biologic DMARDs is associated with risks of infections and malignancies, especially in patients with a weak or compromised immune system. Regular monitoring and vigilance for signs of adverse effects are essential when using biologic agents.^[56]^

##### 3.4.2.3. Corticosteroids.

In cases where NSAIDs alone are insufficient to control inflammation and pain, low-dose glucocorticoids, such as prednisone, may be added to the treatment regimen.^[59]^ They can be administered either through intra-articular injections or systemic administration. Glucocorticoids effectively control inflammation and suppress the immune system, used particularly in managing disease flares and acute inflammation.^[59]^ However, their use should be limited due to potential long-term side effects, including bone loss, weight gain, high blood pressure, skin thinning, infection susceptibility, diabetes, hypertension, sleep disturbances, and mood swings.^[59]^ Prolonged use of corticosteroids can also lead to cataracts, glaucoma, and suppression of the body’s natural corticosteroid production.

##### 3.4.2.4. Emerging therapies.

As research in JIA treatment continues to evolve, new and emerging therapies are being investigated. One promising class of emerging therapies is Janus kinase (JAK) inhibitors, including baricitinib, upadacitinib, and filgotinib. These targeted therapies, recommended by the European League Against Rhematism, can be beneficial for patients who do not respond adequately to other medications,^[55]^ they have shown efficacy in reducing symptoms and disease activity in JIA patients.^[56]^ However, it is crucial to be aware of potential drawbacks associated with JAK inhibitors, including an elevated risk of serious infections, malignancies, and blood clots.^[55,56]^

### 3.5. Prospective advancements in JIA

#### 3.5.1. Utilizing artificial intelligence models for diagnostics and monitoring treatment.

##### 3.5.1.1. Overview.

**Figure 4.**
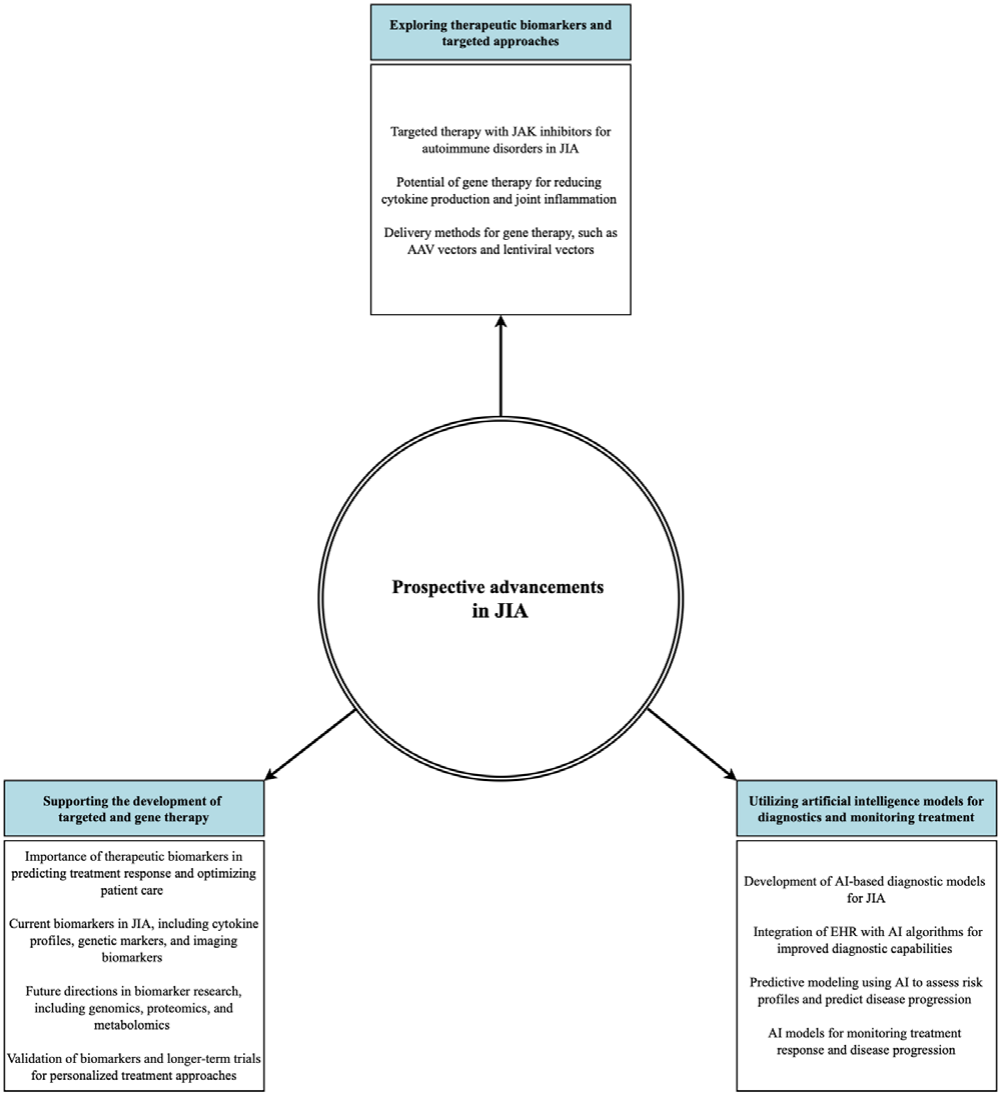
The prospective advancements in JIA. AI = artificial intelligence; EHR, electronic health records; JIA = juvenile idiopathic arthritis.

Artificial intelligence (AI) is a field of computer science that focuses on developing algorithms capable of mimicking human-like intelligent behavior.^[60]^ Within AI, machine learning (ML) is a subfield that explores how computers learn from data.^[61]^ The increasing use of ML in healthcare, especially in predictive and prognostic modeling,^[62]^ holds great potential for improving the diagnosis and treatment monitoring of JIA.^[63]^ A summary of the prospective advancements in JIA can be found in Figure 4.

##### 3.5.1.2. AI-based diagnostic models.

The development and application of AI models for diagnosing JIA have shown promising results. For instance, Nieuwenhove et al (2019) employed a combination of a random forest model and artificial neural networks, achieving a diagnostic accuracy of 90% for JIA. These AI algorithms leverage clinical data, imaging studies, and biomarkers to enhance diagnostic accuracy.^[64]^ By analyzing these diverse data sources, AI-based diagnostic models have the potential to overcome the limitations of traditional diagnostic approaches and aid clinicians in making more accurate diagnoses.

The implementation of electronic health records (EHR) integrated with Natural Language Processing techniques can further augment the diagnostic capabilities of AI models. Peterson et al utilized EHR to develop 103 algorithms, resulting in a positive predictive value of 97% when diagnosing JIA using International Classifications of Diseases Edition 9 and Edition 10 (International Classifications of Diseases-10) codes.^[41]^ By extracting valuable information from text-based data through natural language processing, AI models can leverage EHR to identify patients with JIA more effectively.^[61]^ Integrating EHR with AI algorithms offers greater flexibility and potential for improved patient identification and diagnostic precision.

Another area of exploration involves assessing the risk profile for comorbidities associated with JIA. Through the use of similar techniques, a study investigated the risk profile for chronic uveitis development in individuals with JIA. By employing cluster analysis and data from the Stanford Translational Research Integrated Database Environment, the study achieved a sensitivity of 74% and a specificity of 96% for identifying chronic uveitis.^[65]^ These techniques can be extended to predict genomic risk scores for JIA subtypes, enabling personalized risk stratification.^[66]^ Logistic regression analysis using EHR data from the United Kingdom, United States, and Australia-based cohorts demonstrated the effectiveness of this approach, with an area under the curve ranging from 0.72 to 0.77 for different JIA subtypes.^[66]^ These AI-based predictive models offer valuable insights for disease monitoring and prognosis.

Moreover, AI models can contribute to monitoring treatment response and disease progression in JIA. By analyzing longitudinal data, patient-reported outcomes, and biomarkers, AI algorithms can provide clinicians with valuable information to guide treatment decisions.^[67]^ The Nordic model framework, which employs logistic regression coefficients and data splits, demonstrated its effectiveness in predicting disease course and remission in JIA patients, aiding in personalized treatment planning.^[67]^ Additionally, nonnegative matrix factorization, an unsupervised machine learning technique, identified joint patterns that offer more precise stratification of patients, supporting tailored treatment approaches.^[68]^

##### 3.5.1.3. AI-based treatment monitoring.

The use of AI models for treatment monitoring in JIA holds promise in optimizing drug efficacy and management. By leveraging EHR data and employing the XGBoost algorithm, one study evaluated the efficacy of etanercept, a commonly used JIA treatment. The study identified key predictors of treatment response, achieving a sensitivity of 75%, specificity of 66.67%, and accuracy of 72.22%.^[69]^ This approach enables individualized treatment management based on a patient’s specific response to treatment, improving overall treatment outcomes.

To ensure both feasibility and scientific rigour in clinical trials, a combination of traditional algorithms and machine learning techniques can be employed. Supervised Learning, a subset of machine learning, utilizes labeled data to develop accurate algorithms through classification and training.^[70]^ By utilizing labeled data from rigorous trials, such as those using the Delphi Method for corticosteroid onboarding and tapering, predictive models in arthritis can benefit from the increased predictive value while maintaining academic integrity.^[59]^ Moreover, by stratifying trials based on participants’ genotypes, the delivery of specific medications for drug management can be further personalized, enhancing treatment efficacy.

The integration of AI models into the diagnostic process and treatment monitoring of JIA holds great potential for improving patient outcomes and advancing our understanding of this complex condition. With the ability to analyze diverse data sources and extract valuable insights, AI can aid in accurate diagnosis, risk stratification, treatment decision-making, and personalized treatment management in JIA. Continued research and implementation of AI-based approaches are essential to harness the full potential of these advancements in JIA care.

#### 3.5.2. Exploring therapeutic biomarkers and targeted approaches in JIA.

##### 3.5.2.1. Overview.

Therapeutic biomarkers play a vital role in JIA research by facilitating the identification of effective drug targets and predicting treatment response. These biomarkers serve as measurable indicators of drug therapy’s effects, enabling personalized and targeted treatment approaches. Understanding the concept and significance of therapeutic biomarkers in JIA research provides valuable insights into the disease and improves treatment outcomes.

Identifying biomarkers capable of predicting treatment response is crucial in JIA research. These biomarkers act as early indicators of disease progression, severity, and response to specific treatments. Analyzing these markers enables clinicians to make informed decisions about treatment strategies, optimizing patient care while minimizing the risk of adverse effects associated with ineffective treatments.

##### 3.5.2.2. Current biomarkers in JIA.

Several biomarkers are currently under investigation in the context of JIA research, including cytokine profiles, genetic markers, and imaging biomarkers. Cytokine profiles, such as IL-6 and TNF, have demonstrated potential utility as biomarkers in JIA. Studies have shown that elevated levels of specific proinflammatory proteins, such as myeloid-related S100 proteins (MRP8/14), can predict disease progression, severity, and treatment response in JIA.^[71,72]^ For example, concentrations of S100A12 above 213 ng/ml were found to predict a minimum ACRPedi50 response, with subsequent reductions after 4 weeks of etanercept treatment.^[73]^ Additionally, higher MRP8/14 levels before initiating methotrexate treatment correlated with a higher likelihood of treatment response at the 6-month follow-up.^[74]^

Genetic markers, including HLA-B27, show promise in predicting treatment efficacy in JIA. Increased presence of HLA-B27 has been associated with a greater likelihood of not achieving clinical remission over an 8-year period.^[75]^ Studies investigating the relationship between interleukin 1 receptor antagonist (IL1RN) alleles and anakinra therapy have found that higher expression of IL1RN alleles in JIA patients strongly correlated with a lack of response to anakinra.^[76]^ These genetic biomarkers provide valuable insights into individual patients’ likelihood of responding to specific treatments.

Imaging biomarkers obtained through techniques such as magnetic resonance imaging and ultrasonography offer noninvasive means of assessing disease activity and treatment response in JIA. They allow clinicians to visualize joint inflammation, cartilage damage, and synovial hypertrophy, providing objective measurements for evaluating treatment effectiveness.

##### 3.5.2.3. Future directions in biomarker research.

The field of biomarker research in JIA is continually evolving, with emerging biomarkers and novel techniques opening new avenues for exploration. Omics technologies, including genomics, proteomics, and metabolomics, hold immense potential for identifying novel drug targets and developing personalized treatment approaches.

Genomics studies can unravel the genetic variations underlying JIA, providing insights into disease mechanisms and potential therapeutic targets. Proteomics focuses on analyzing proteins and their modifications. By studying the proteome of JIA patients, researchers can identify specific protein biomarkers indicative of disease activity and treatment response. Metabolomics offers a comprehensive view of the metabolic alterations associated with JIA, identifying metabolite biomarkers that provide insights into disease mechanisms and potential therapeutic targets.

While technological advancements in biomarker identification hold promise for improving drug efficacy in JIA, further research is necessary. Studies involving larger populations and longer trial durations are required to validate the efficacy of biomarkers and establish their utility in guiding personalized treatment approaches for JIA.^[77]^

Overall, exploring therapeutic biomarkers in JIA research enhances treatment outcomes and optimizes patient care. Leveraging existing and emerging biomarkers provides valuable insights into disease mechanisms, identification of appropriate drug targets, and personalization of treatment strategies, ultimately improving the quality of life for individuals living with JIA.

#### 3.5.3. Supporting the development of targeted and gene therapy in JIA.

##### 3.5.3.1. Targeted therapy for autoimmune disorders in JIA.

Targeted therapies have emerged as a promising area of research for treating autoimmune disorders, including JIA. These therapies aim to suppress specific molecules or pathways implicated in JIA, minimizing unwanted side effects by targeting specific components rather than the entire immune system. An example of targeted therapy in JIA is the use of JAK inhibitors, which target JAK family enzymes involved in inflammation and autoimmune disorders.^[78]^ By blocking proteins in the JAK signaling pathway, which is activated by cytokines such as interleukins, JAK inhibitors effectively reduce inflammation and help control JIA symptoms. Clinical trials investigating JAK inhibitors, including tofacitinib, baricitinib, and upadacitinib, have demonstrated a reduction in joint pain and swelling in children with polyarticular JIA.^[56,79]^ Further research and trials targeting different JIA-associated compounds are warranted to advance the field of targeted therapy.

##### 3.5.3.2. Gene therapy for JIA.

Gene therapy holds great potential for JIA treatment by introducing new or modified genes into the body to correct or replace defective genes involved in the disease. In JIA, gene therapy aims to reduce cytokine production and joint inflammation, improving symptoms and slowing down disease progression. Although significant advancements in gene-based therapy for JIA are yet to be achieved, alternative delivery methods have been explored to facilitate targeted therapy.^[80,81]^

Delivery methods such as adeno-associated virus vectors, lentiviral vectors, messenger ribonucleic acid therapy, and ex vivo gene therapy have been investigated in JIA research.^[80,81]^ The choice of delivery method depends on the specific gene or protein being delivered and the underlying mechanisms of the disease. For example, adeno-associated virus vectors have been utilized to deliver modified interleukin-6 (IL-6) receptor genes, effectively reducing joint inflammation and improving symptoms in animal studies.^[82]^ Lentiviral vectors have shown promise in delivering anti-inflammatory genes such as interleukin-10 (IL-10), interleukin-1 receptor antagonist (IL-1RA), TNFAIP3, and TNF receptor superfamily member 12A (TNFRSF12A), regulating the immune response and reducing inflammation, leading to reduced joint damage and improved symptoms.^[83]^ messenger RNA therapy, involving the direct delivery of anti-inflammatory proteins into the body without the need for a functional gene, has shown potential as a faster and more efficient approach for JIA gene therapy.^[83,84]^ Another approach, ex vivo gene therapy, involves modifying T-cells outside the body to express anti-inflammatory genes and then reintroducing them into the body.^[85,86]^ However, further studies are needed to validate and generalize these findings on a larger scale.

##### 3.5.3.3. Targeting B-cells in JIA.

B-cells, a type of white blood cell, play a critical role in the immune response and antibody production to protect the body against infection and disease. In JIA, B-cells can contribute to joint inflammation and damage through antibody production. Therefore, targeting B-cells has emerged as a potential therapeutic strategy for JIA.

Several approaches are being explored to target B-cells in JIA, including B-cell depletion therapy, B-cell-directed immunotherapy, and anti-B cell antibodies. B-cell depletion therapy involves the use of drugs like Rituximab and Ocrelizumab, which specifically target and destroy B-cells, thereby reducing the production of antibodies that contribute to joint inflammation and damage.^[87]^ B-cell-directed immunotherapy aims to target the signaling pathways involved in B-cell activation and function, leading to a reduction in B-cell activity and antibody production. Drugs such as Tofacitinib and Baricitinib have been employed in B-cell-directed immunotherapy.^[2]^ Furthermore, anti-B cell antibodies, such as Belimumab and Ofatumumab, have been specifically designed to neutralize B-cells, effectively reducing the production of antibodies that contribute to joint inflammation and damage in JIA.

By exploring these various strategies for targeting B-cells, researchers aim to develop effective therapies that mitigate the harmful effects of B-cell-mediated inflammation in JIA. The development of targeted and gene therapy approaches holds great promise for JIA treatment. Further research, clinical trials, advancements in delivery methods, and therapeutic strategies are essential to realizing the full potential of these treatment modalities in improving patient outcomes and quality of life.

#### 3.5.4. Biomechanics and 3D printing to improve quality of life.

##### 3.5.4.1. Physical and occupational therapy for joint improvement.

Physical rehabilitation with non-pharmacological interventions plays a crucial role in improving the quality of life for patients diagnosed with JIA. Early implementation of physical therapy has been shown to enhance joint function, mobility, range of motion, muscle strength, and endurance.^[88,89]^ Furthermore, physical therapy can effectively reduce pain and fatigue in JIA patients. Occupational therapy also holds significance in assisting individuals with autoimmune conditions, enabling them to perform daily activities while minimizing stress on affected joints.^[90]^ Both physical and occupational therapy interventions encompass exercises to enhance flexibility, strength, balance, fine motor skills, and coordination. Although muscle soreness and fatigue may arise as side effects, these therapies are generally considered safe.^[90,91]^ In cases where joint damage persists despite conservative treatments, surgical options such as joint fusion, joint replacement, and arthroscopy may be necessary.

##### 3.5.4.2. Biomechanical insights into JIA gait disturbances.

Prolonged JIA can result in gait disturbances characterized by a slower gait velocity and decreased range of motion at the hip, knee, and ankle joints.^[92]^ These disturbances arise from the inflammatory and destructive processes occurring within the joints of JIA patients. The field of biomechanics offers new possibilities for treatment planning and prognostic testing in JIA patients. Montefiori et al (2009) conducted a study utilizing a musculoskeletal model created from magnetic resonance images to predict joint contraction forces (JCF) and assess the impact of joint impairment on 18 children with JIA. Their findings indicated that knee overloading during the gait cycle may serve as a predictor of disease progression.^[93]^ This example highlights the potential of biomechanical assessment in stratifying JIA patients and guiding the development of individualized management plans, ultimately leading to improved patient outcomes.

##### 3.5.4.3. Harnessing the potential of 3D printing technology.

The advent of 3D printing technology holds promise for assessing disease progression and facilitating joint regeneration in JIA. Remarkable advancements in 3D printing have made it a feasible and high-quality modeling tool. Kleyer et al (2014) utilized CT images to generate 3D printed metacarpal heads, enabling precise location and measurement of cortical breaks resembling bone erosions.^[94]^ These models were created based on one patient with rheumatoid arthritis (RA) and a healthy control, showcasing the potential application of this technology in monitoring joint destruction progression and evaluating post-directed injection improvements.^[94]^ Additionally, 3D bioprinting technology has facilitated the development of sophisticated scaffolding models that can be implanted into joints, containing biomaterials capable of promoting joint regeneration.^[95]^ This approach presents a potential future therapeutic option for patients with arthritic diseases, including JIA, allowing for natural lesion healing and potentially reducing the need for surgical interventions.

##### 3.5.4.4. Cutting-edge studies and future directions.

To further enhance the understanding and application of biomechanics and 3D printing in JIA, ongoing studies are investigating novel techniques and approaches. For instance, Salchow-Hömmen et al (2022) explored the use of portable technology to assess gait patterns and joint biomechanics in JIA patients, yielding valuable insights into disease progression and treatment response.^[96]^ Furthermore, Meng et al (2022) demonstrated the successful fabrication of patient-specific 3D-printed joint replacements in a cohort of JIA patients, highlighting the potential of personalized and regenerative approaches.^[97]^ Meng et al, (2022) Continued research and development in these areas hold great promise for advancing the field and ultimately improving the quality of life for individuals with JIA.^[97]^

## 4. Conclusion

JIA is a multifactorial condition that has progressed in its research regarding its pathophysiology. With the improvements in genetics research, targeted therapies may take place for children presenting with JIA. The long-term consequences of gait instability, chronic joint pain, and nonadherence to treatment due to its side effects should be the forefront of JIA research. Developments in AI, genetic therapies, molecular biomarkers, and 3D printing can aid in increasing the quality of life for children suffering from different JIA subtypes. However, future investigations should explore these therapeutic options allowing individualized treatment and personalized medicine.

## Acknowledgments

We would like to thank the Inter-Continental Omni-Research in Medicine (ICORMed) Collaborative members for their contributions and support for this manuscript.

## Author contributions

**Conceptualization:** Helen Ye Rim Huang, Andrew Awuah Wireko, Goshen David Miteu.

**Data curation:** Helen Ye Rim Huang, Andrew Awuah Wireko, Goshen David Miteu.

**Formal analysis:** Helen Ye Rim Huang.

**Methodology:** Helen Ye Rim Huang.

**Supervision:** Helen Ye Rim Huang, Andrew Awuah Wireko, Marios Papadakis.

**Validation:** Helen Ye Rim Huang.

**Visualization:** Helen Ye Rim Huang.

**Writing – original draft:** Helen Ye Rim Huang, Andrew Awuah Wireko, Goshen David Miteu, Adan Khan, Sakshi Roy, Tomas Ferreira, Tulika Garg, Narjiss Aji, Faaraea Haroon, Farida Zakariya, Yasir Alshareefy, Anushka Gurunath Pujari, Djabir Madani.

**Writing – review & editing:** Helen Ye Rim Huang, Andrew Awuah Wireko, Goshen David Miteu, Adan Khan, Sakshi Roy, Tomas Ferreira, Tulika Garg, Narjiss Aji, Faaraea Haroon, Farida Zakariya, Yasir Alshareefy, Anushka Gurunath Pujari, Djabir Madani, Marios Papadakis.
